# Jingqianshu granules mitigates premenstrual depression by regulating orexin signaling

**DOI:** 10.3389/fphar.2024.1294122

**Published:** 2024-06-14

**Authors:** Ping Dong, Weibo Dai, Tingting Zhao, Yuandong Gong, Ning Weng, Shimeng Lv, Yifan Zhao, Chunyu Du, Yuexiang Ma, Zhen Zhang, Shuhua He, Feng Zheng, Peng Sun

**Affiliations:** ^1^ School of Traditional Chinese Medicine, Shandong University of Traditional Chinese Medicine, Jinan, China; ^2^ Department of Pharmacy, Zhongshan Hospital of Traditional Chinese Medicine, Zhongshan, China; ^3^ School of Foreign Language, Shandong University of Traditional Chinese Medicine, Jinan, China; ^4^ Shandong Mental Health Center, Jinan, China; ^5^ Department of First Clinical Medical College, Shandong University of Traditional Chinese Medicine, Jinan, China; ^6^ School of Pharmacy, Shandong University of Traditional Chinese Medicine, Jinan, China; ^7^ Qinhuangdao Shanhaiguan Pharmaceutical Co., Ltd., Qinhuangdao, China; ^8^ College of Traditional Chinese Medicine Health, Guizhou University of Traditional Chinese Medicine, Guiyang, China; ^9^ Boai Hospitai of Zhngshan, Zhngshan, China; ^10^ Department of Neurosurgery, The Second Affiliated Hospital of Fujian Medical University, Quanzhou, China; ^11^ Innovative Institute of Chinese Medicine and Pharmacy, Shandong University of Traditional Chinese Medicine, Jinan, China

**Keywords:** Jingqianshu granules, premenstrual, orexin, OX1R, OX2R, inflammatory factor

## Abstract

**Introduction:** Premenstrual dysphoric disorder (PMDD), a severe form of premenstrual syndrome (PMS), is a serious health disorder that affects patient moods. It is caused by cyclic psychological symptoms and its pathogenesis is still unclear. Abnormalities in the basolateral amygdala (BLA) orexin system, which are important causes of the development of depressive mood, have not been reported in PMDD, so exploring its intrinsic mechanisms is meaningful for enriching the pathomechanisms of PMDD.

**Methods:** High performance liquid chromatography was used for the determination of the active ingredients of Jingqianshu granules. Developing a rat model of premenstrual depression using the forced swimming test (FST). The experiment consisted of two parts. In Part 1, the rats were divided into the control group, the model group, the model + Jingqianshu group, and the model + fluoxetine group. The FST, open field test, and elevated plus maze test, were used to assess the behavior of the rats as well as to evaluate the effect of drug intervention. Immunofluorescence and RT-qPCR were used to detect the expression of orexin and its receptors OX1R and OX2R genes and proteins. The expression of Toll-like receptor 4, nuclear factor kappa-B, tumor necrosis factor-α, interleukin 6, and interleukin-1β in the BLA brain region was detected by Western-Blot. In part 2, the rats were injected intracerebrally with orexin-A. Observe the behavioral activities of rats in the control group, model group, and model+orexin-A group. Immunofluorescence was used to detect microglia in the BLA area of rats, and the expression levels of the above inflammatory factors were detected by Western-Blot.

**Results:** The five components of Jingqianshu granules are: paeoniflorin, erulic acid, liquiritin, hesperidin, and paeonol. During the estrous cycle, rats exhibited depressive-like behavior during the non-receptive phase of the behavioral test, which disappeared during the receptive phase. Immunofluorescence and RT-qPCR showed reduced gene and protein expression of orexin, OX1R, and OX2R in the BLA region of rats in the model group.WB showed elevated levels of inflammatory factors. All returned to control levels after drug treatment. In part 2, injection of orexin-A into the BLA brain region of model rats resulted in reduced immunoreactivity of microglia and decreased expression levels of inflammatory factors.

**Discussion:** Jianqianshu granules can achieve the purpose of treating premenstrual depression by regulating orexin-mediated inflammatory factors, which provides a new idea for further research on the pathogenesis of PMDD. However, the current study is still preliminary and the pathogenesis of PMDD is complex. Therefore, more in-depth exploration is needed.

## Introduction

Premenstrual dysphoric disorder (PMDD), a severe form of premenstrual syndrome (PMS), is a condition in which women of a childbearing age exhibit cyclic discomfort, mood, and physical disturbance symptoms during the luteal phase, includes depressive and anxiety-like mood changes, and locomotor activity alterations (Mishra et al., 2023). In particular, the depressed mood of the patient severely affects the ability to learn, work, and the quality of life, which adds to the social burden (Tiranini and Nappi, 2022). However, the exact mechanisms involved are not clear and are generally considered to be a consequence of multiple factors. Currently, first-line therapeutic drugs that target 5-hydroxytryptamine transporter, such as the serotonin reuptake inhibitors (SSRIs) in PMDD have shown limited efficacy and high side effects in clinical practice (Marjoribanks et al., 2013). In a study of antidepressant medication adherence for the treatment of PMS/PMDD (Sundström-Poromaa et al., 2000), it was noted that 101 patients treated with SSRIs for several months to more than 2 years experienced the following adverse effects: Reduced libido (45%), Dysorgasmia (28%), Nausea (22%), Weight gain (20%), Sweatings (19%), Headache (16%), Vertigo (14%), Sleeping disorders (12%). In addition, alternative therapies, such as behavioral cognitive therapy (Weise et al., 2019), herbal medicine (Xu et al., 2020), and acupuncture (Armour et al., 2018) have been gaining widespread attention, Jingqianshu granules have been recognized for their clinical efficacy (Zhongqiu, 2016).

Orexin (also known as hypocretin) is a recently discovered hypothalamic neuropeptide that includes hypocretin-A/orexin -A and hypocretin-B/orexin -B. Its receptors include type 1 HcrtR1/OX1R and type 2 HcrtR2/OX2R (Sakurai et al., 1998; de Lecea et al., 1998; Li et al., 2014). Orexin is encoded by prepro-orexin mRNA and then cleaved by protein hydrolysis to form orexin-A and orexin-B, the former with 33 amino acid residues and two intramolecular disulfide bridges in its N-terminal structural domain, and the latter with 28 amino acid residues. Orexin-A and orexin-B share 46% sequence homology (Tsunematsu and Yamanaka, 2012). Orexin peptides are highly conserved in vertebrates, especially in mammals: orexin-A has 100% sequence homology, while orexin-B differs only by 1 or 2 amino acids between species (Jacobson et al., 2022). Orexin-A has an equally high affinity for OX1R and OX2R, while orexin-B has approximately 10-fold selectivity for OX2R (Pizza et al., 2014). Studies have shown that orexin is expressed in many brain regions, with the amygdala receiving orexinergic fibers originating from the lateral hypothalamus and expressing OX1R (Pan et al., 2020). The BLA, which is the inner subnucleus of the amygdala, plays a key role in the regulation of depression and behavior (Grogans et al., 2022). There is evidence that the amygdala shows estrous cycle-related activity in emotional memory (Blume et al., 2017) and that the major affective disorders more commonly seen in women (e.g., depression, anxiety, posttraumatic stress disorder) are associated with excessive activation of the BLA (Victor et al., 2010). A meta-analysis (Etkin and Wager, 2007) of a neuroimaging study of women aged 18–55 years noted that women showed greater activation of negative emotions in the left amygdala than did men, it is consistent with the results of Sheline et al. for functional magnetic resonance imaging studies, where the left amygdala was significantly more activated in depressed patients than in controls (Sheline et al., 2001). Recent studies have found that orexin and its receptors can modulate the inhibitory and excitatory effects of BLA neurons (Gyawali and James, 2022), but this has not been reported in PMDD. In addition, research has shown that orexin is a promising target for the treatment of depression, that orexin dysregulation is an important mechanism for the onset of depressive symptoms (Khairuddin et al., 2020) and that orexin levels vary with depression-like behavior (Nocjar et al., 2012). Moreover, studies have demonstrated a high degree of concordance between PMDD and major depressive disorders (MDD) at the macroscopic level, including onset, symptoms, and population, and at the microscopic level, as shown in internal pathogenesis studies. The co-morbidity rate of PMDD and MDD ranged from approximately 30%–70% (Klatzkin et al., 2010). Therefore, we hypothesized that orexin has the same potential role in PMDD and that modulation of orexin-A in BLA could be effective in treating premenstrual depression.

In addition, orexin has been shown to have a neuroimmune role, and Zhang et al. found that orexin-A was able to suppress endothelial cell inflammation by inhibiting MAPK p38 and NF-κB inflammatory signaling pathways (Zhang et al., 2018). In an experiment, Sun et al. found that orexin-A treatment reduced the secretion of interleukin 1β (IL-1β), interleukin 6 (IL-6), and interleukin 8 (IL-8) and the production of reactive oxygen species (ROS), and inhibited the activation of TNF-α-induced nuclear factor-κB (NF-κB) signaling pathway (Sun et al., 2018). Emerging trends in neuroendocrinology link inflammatory processes to mental and somatic disorders with common features of PMS/PMDD, low-grade inflammation is associated with changes in mood and pain (Puder et al., 2006), and inflammatory markers in women are associated with the degree of menstruation and premenstrual symptoms and are expressed at higher levels than in healthy women (Bertone-Johnson et al., 2014). Therefore, the relationship between orexin and inflammation needs to be further investigated.

Jingqianshu granules are made of 11 Chinese herbs, including White peony, Bupleuri Radix, Angelica, Atractylodes, Moutan Cortex, Rhizoma Cyperi, Tangerine peel, Turmeric Root Tuber, Amomi Fructus, Ginseng, and Liquorice. It has the effect of relieving depression and relieving pain. It is mainly used for the treatment of premenstrual depression and melancholy (Zhang and Ma, 2010; Su and Xue, 2011). In this study, ovariectomized rats exposed to repeated hormone injections and FST tests were used as a PMDD model (Wei et al., 2020). The effects of Jingqianshu granules on behavior and the expression of orexin-A and orexin receptors in BLA brain regions were assessed, and the coordinated role of orexin and inflammatory factors in the disease was further investigated.

## Experimental materials and methods

### UHPLC-PDA measurement of Jingqianshu granules

The Jingqianshu granules were analyzed using UHPLC (Waters e 2965, United States). After preparation of the test solution of Jingqianshu 10 μL was taken and injected into the liquid chromatograph and the chromatogram was recorded for 120 min. A Waters Symmetry C18, 250 mm × 4.6 mm, 5 μm column was used for all analyses. The flow rate was 1.0 mL/min, the detection wavelength was 292 nm, and the column temperature was 30°C. The mobile phase was a mixture of acetonitrile (A) and 0.1% phosphoric acid (B). The samples were scanned at a full wavelength from 200 to 400 nm using a PDA detector, and the absorbance maps were analyzed.

### Animals

One hundred female Wistar rats weighing 180 ± 15 g at 6–8 weeks of age were purchased from Jinan Pengyue Experimental Animal Breeding Co., Ltd., Jinan, China. The rats were placed at an ambient temperature of 21°C ± 1°C and 55% relative humidity with a 12 h/12 h light/dark cycle (light on at 20:00; light off at 8:00). All procedures were performed under dim light (<25lux), with food and water available *ad libitum*. The animals were allowed to acclimatize for a week before the experiment. The animal study was reviewed and approved by the Ethics Review Committee of Shandong University of Traditional Chinese Medicine [No.: SDUTCM20210806003].

### Ovariectomy

Ovariectomy was performed on all rats. The rats were anesthetized with 2% sodium pentobarbital (60 mg/kg) intraperitoneally and operated under aseptic conditions. The skin of the rat’s abdomen was cut open (1 cm wide) with a scalpel to expose the abdominal cavity. The ovaries were searched for in the midline of the rat abdomen on both sides, and bilateral tubal ligation and removal of the ovaries were performed. Finally, the incision was sutured and a few drops of penicillin were applied to the wound. Penicillin injection was administered daily and the rats were allowed to recover for 3 days while maintaining the original feeding conditions.

### Hormone initiation program

Studies have shown that the rat estrous cycle consists of a receptive phase (proestrus/estrus) and a non-receptive phase, with the non-receptive phase consisting of diestrus 1, diestrus 2, and metestrus, each lasting approximately 1 day (Ho et al., 2001). After wound healing, exogenous estrogen and progesterone were administered to induce cyclic estrus in the rats to establish a regular estrous cycle: 0.5 µg estradiol benzoate (Hefei Xinkexin Animal Pharmaceutical Company, Hefei Anhui,20220201) was injected into the first day as phase D1, 0.5 µg estradiol (Shanghai Macklin Biochemical Co., Ltd., Shanghai, China, C13752449) on the day of phase D2, and 0.5 mg of progesterone (Hefei Xinkexin Animal Pharmaceutical Company, Hefei Anhui,20220201) on the day of P/E phase. These hormones were dissolved in 0.1 mL of dimethyl sulfoxide and injected subcutaneously at 12:00 daily (Wei et al., 2020). For the second cycle of the hormone pre-stimulation protocol, FST was performed on the day of the D1 and P/E phases, and the hormone stimulation protocol was continued until the end of the experiment.

### Forced swimming test

The FST was used to establish a model of premenstrual depression in rats and depression-like behavior was assessed (Castagné et al., 2011). The rats were placed in a plexiglass cylinder with a diameter of 20 cm, a water depth of 30 cm, and a temperature of 23°C for 5 min. The cumulative duration of immobility and the latency of immobility (time from the beginning of the experiment to first immobility) were calculated. Immobility was defined as the absence of movement except for that necessary to keep the nose above the water surface for breathing. After each test, the water was changed and the cylinders were carefully cleaned. This test was performed between 12:00 and 16:00.

### Grouping

**FIGURE 1:**
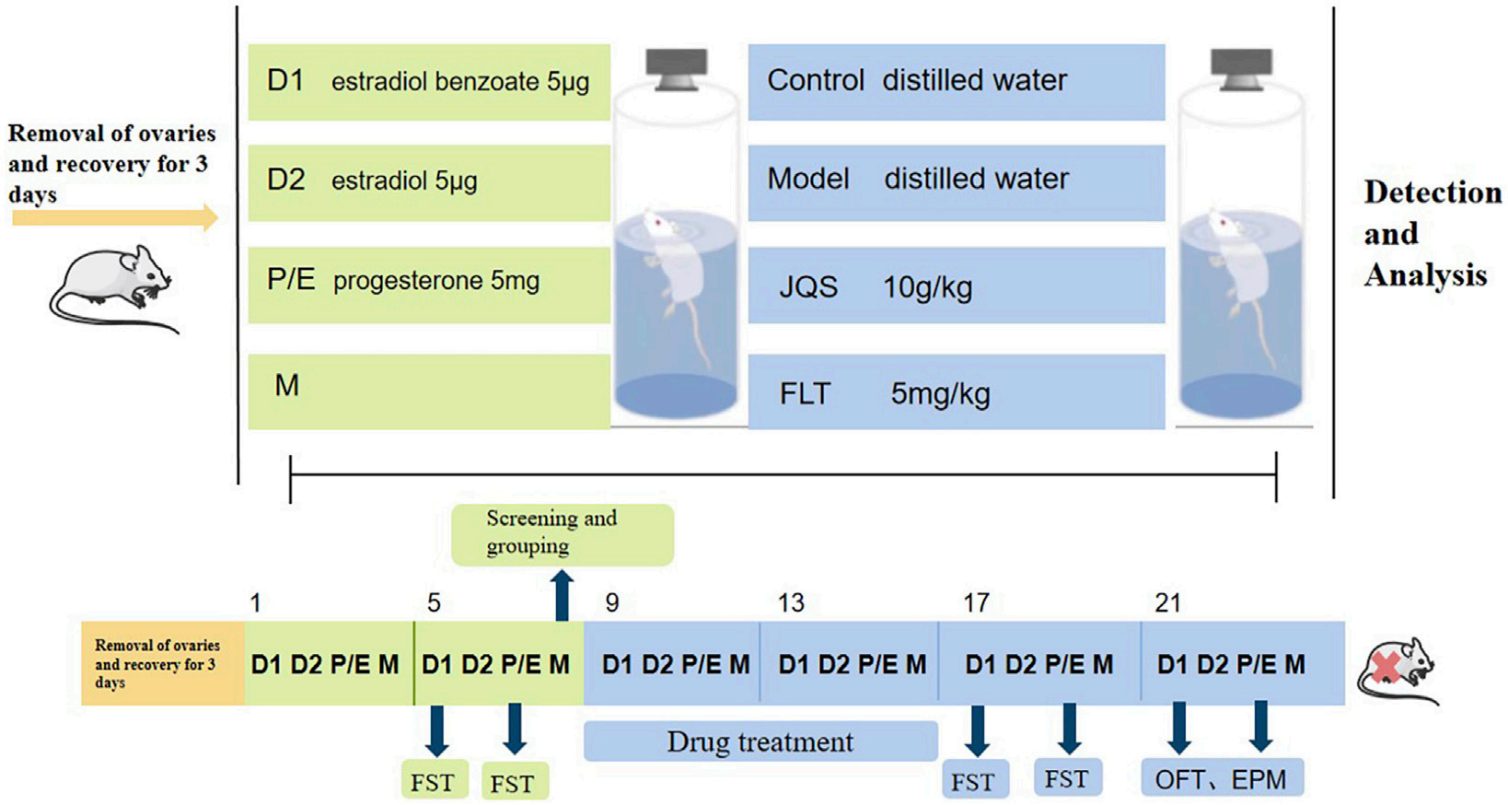
The first part of the animal experimental protocol of this study. D1, Diestrus 1 phase; D2, Diestrus 2 phase; P/E, Proestrus/Estrus phase; M, Metestrus phase; FST, Forced swimming test.

**FIGURE 2:**
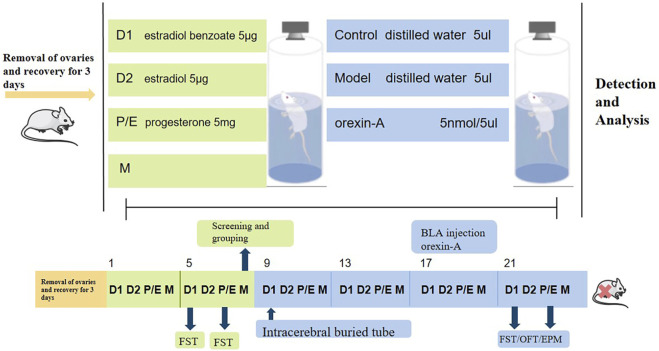
The second part of the animal experimental protocol of this study. D1, Diestrus 1 phase; D2, Diestrus 2 phase; P/E, Proestrus/Estrus phase; M, Metestrus phase; FST, Forced swimming test.

The experiment is divided into two parts. FST data were collected from rats in the non-receiving D1 and receiving P/E phases, and the rats were ranked from highest to lowest based on the differences between the immobility duration of the two phases. In Part 1 of the experiment (Figure 1), the top 30% of rats were randomly divided into the model group, model + Jingqianshu granules group (model + JQS) and model + fluoxetine group (model + FLT), while the bottom 10% of rats were assigned to the control group. Each group contains 10 rats. In Part 2 of the experiment (Figure 2), the former 20% of the rats were randomly divided into model and model + orexin-A groups, while the last 10% of the rats were the control group, each group contains 10 rats.

### Drug treatment

From day 9 to day 16, rats in the model + JQS group were given Jingqianshu granules (10 g/kg) (Zhang and Ma, 2010) and rats in the model + FLT group were given fluoxetine (5 mg/kg) (Gómez et al., 2014). Jingqianshu granules (batch number: 20220501, Shanhaiguan Pharmaceutical Co., LTD, Qinhuangdao, Hebei) and fluoxetine dispersible tablets (batch number:220113, Shandong Linuo Pharmaceutical Company, Jinan, Shandong) were dissolved in distilled water and administered intragastrically (2 mL) at 08:00 daily. Rats in the control and model groups received the same volume of distilled water. After drug treatment, FST was performed on day 17 (D1) and day 19 (P/E), respectively.

### BLA brain area injection of orexin-A

The rat was anesthetized with 2% pentobarbital sodium, placed in a prone position, the head was fixed on a brain stereotaxic apparatus, the top of the head was shaved, sterilized with alcohol, and a 0.5–1.0 cm incision was made in the center of the head to expose to the skull, and the BLA position was determined according to the rat stereotaxic atlas: AP2.8 mm, RL5. 0 mm, H8.6 mm, and labeled. A dental drill is used to drill through the marker point to the dura mater, the dura mater is punctured with a needle, and the catheter with inner core is punctured to a depth of 4.5 mm. The puncture cannula was fixed to the skull with self-coagulating toothrest powder, and the incision was sutured. After the surgery, penicillin was given intramuscularly (100,000 units/kg once daily) for 3 days to prevent intracranial infection. Recovery 7 days after surgery, the blood-brain barrier was restored, and the model + orexin-A group was given orexin-A (Glpbio, Shanghai, China) in the BLA brain region for four consecutive days at a dose (Han et al., 2022) of 5 nmoL/5 μL (dissolved in saline); the control group was given saline 5 μL. In the awake state of the animal, the inner core of the catheter was pulled out, and the inner needle was inserted into the destination site with a micro pump to inject drug or solvent (1 μL/min), and the inner core was inserted again to close the tube.

### Open field test

The open field test was performed after drug treatment (on days 21 and 23 of the experiment). In a quiet environment, a faint red light (<12lux) was turned on and the rats were placed at the center of a black open field box (100 cm × 100 cm) with their backs turned to the researcher and allowed to move freely. The area of the open field box was divided into nine grids, with the central area occupying 1/9 of the entire open field and the rest being the peripheral area. SuperMaze+ high-throughput animal behavior analysis software was used to record and track the total distance traveled by the rats, average roaming speed, and time to enter the central area over 5 min.

### Elevated plus maze test

On days 21 and 23 of the experiment, 2 open arms (10 cm × 50 cm) and 2 closed arms (10 cm × 50 cm) were used. The apparatus was elevated 76 cm above the ground and the rats were placed on the central platform with their heads facing the outstretched arms. The behavior of the rats was recorded for 5 min using the SuperMaze+ high-throughput tracking system. The number of open arm entries (OE), number of closed arm entries (CE), time to enter the open arm (OT), and time to enter the closed arm (CT) of the rats were analyzed. Based on these results, OT% and OE% were calculated according to the following equations: OT% = OT/ (OT + CT) × 100%; OE% = OE/ (OE + CE) × 100%.

### Sample collection

At the end of the behavioral experiment, the rats were anesthetized with 2% sodium pentobarbital (200 mg/kg), and BLA tissue was removed, dispensed into cryopreservation tubes, sealed with parafilm, and stored at −80°C until processed. The entire brains of the remaining rats in each group were fixed in 4% paraformaldehyde (PFA) solution and prepared for tissue sectioning.

### Immunofluorescence staining

Paraffin sections were obtained from the brain tissue of each rat. The sections were stained with anti-orexin-A, anti-OX1R, and anti-OX2R, respectively. The process involved in immunofluorescence staining is briefly described as follows: first, the paraffin sections were dewaxed and rehydrated, then the antigens were extracted, circulated, and blocked with endogenous peroxidase, and then blocked with serum. The slides with the first primary antibody (appropriately diluted with PBS) were incubated overnight at 4°C and placed in a wet box containing a small amount of water. Then, the samples were washed with PBS for 3 min × 5 min and incubated with the corresponding secondary antibodies. Self-fluorescent bursting agents were added and the nuclei were inhibited with DAPI (G1012; Servicebio; Wuhan, China) and covered with anti-fading fluorescent fixation medium (G1401; Servicebio; Wuhan, China). Finally, the sections were observed with a fluorescence microscope (Nikon Eclipse C1, NIKON, Japan) and with ImageJ software to analyze the mean fluorescence intensity.

### Reverse transcription PCR and quantitative real-time PCR

Total RNA was isolated using the RNA Rapid Extraction Kit (Servicebio, G3013) and reverse transcribed into cDNA using the Revert Aid First Strand cDNA Synthesis Kit (Servicebio, G3330) and was then amplified using PCR with specific primers (Supplementary Table S1). The reaction products were separated using electrophoresis and images were captured using a gel image analysis system (Bio-Rad, United States). The intensity of the bands was analyzed using Image Pro Plus 6.0 software and the values were normalized to that of GAPDH. Quantitative real-time PCR was performed using a Bio-Rad IQ5 real-time PCR system (Bio Rad, United States), while relative fold changes in mRNA expression were determined using the 2-(ΔΔCt) method. GAPDH was used as the loading control.

### Western blot

The rat tissue blocks were washed 2-3 times with pre-cooled PBS to remove blood stains, cut into small pieces, and placed in homogenization tubes, two 4 mm homogenization beads were added, 10 times the volume of tissue lysate was added, and the homogenization program was set to homogenize. After completion, the homogenization tube was removed and placed on ice in lysis solution for 30 min, shaking every 5 min to ensure complete lysis of the tissue. Then centrifuge at 12000 rpm, 4°C, for 10 min and collect the supernatant, which is the total protein solution. Total protein concentrations were determined using a BCA kit (G2026; Servicebio; Wuhan, China) according to the manufacturer’s instructions. Proteins were subjected to sodium dodecyl sulfate-polyacrylamide gel electrophoresis (SDS-PAGE) (G2003-50T; Servicebio; Wuhan, China), transferred to polyvinylidene difluoride (PVDF) membranes, blocked with 5% bovine serum albumin (BSA) for 1 h, and incubated with primary antibody at 4°C overnight. The primary antibodies used were as follows: ACTIN (GB15003; Servicebio; Wuhan, China); TNF-a (CST; Servicebio; Wuhan, China); TLR-4 (GB11519; Servicebio; Wuhan, China); NF-κB p65 (GB12142; Servicebio; Wuhan, China); IL-6 (GB11117; Servicebio; Wuhan, China); IL-1β(GB11113; Servicebio; Wuhan, China); The secondary antibody used was HRP-goat anti-rabbit (GB23303; 1:5000; Servicebio; Wuhan, China). Finally, the strips are displayed by chemiluminescence and the optical density values of the target strips are analyzed by the Image software processing system.

### Statistical analysis

The above experimental data were analyzed and plotted using GraphPad Prism 8 software. Behavioral data using a two-way ANOVA followed by a *post hoc* Sidak multiple comparison test. For neurochemical data, used one-way ANOVA and *post hoc* Tukey’s multiple comparison test. The results are expressed as mean ± standard error (mean ± SEM) with *p* < 0.05 indicating a statistical difference between the groups.

## Results

### Quality control of the Jingqianshu granules

**FIGURE 3:**
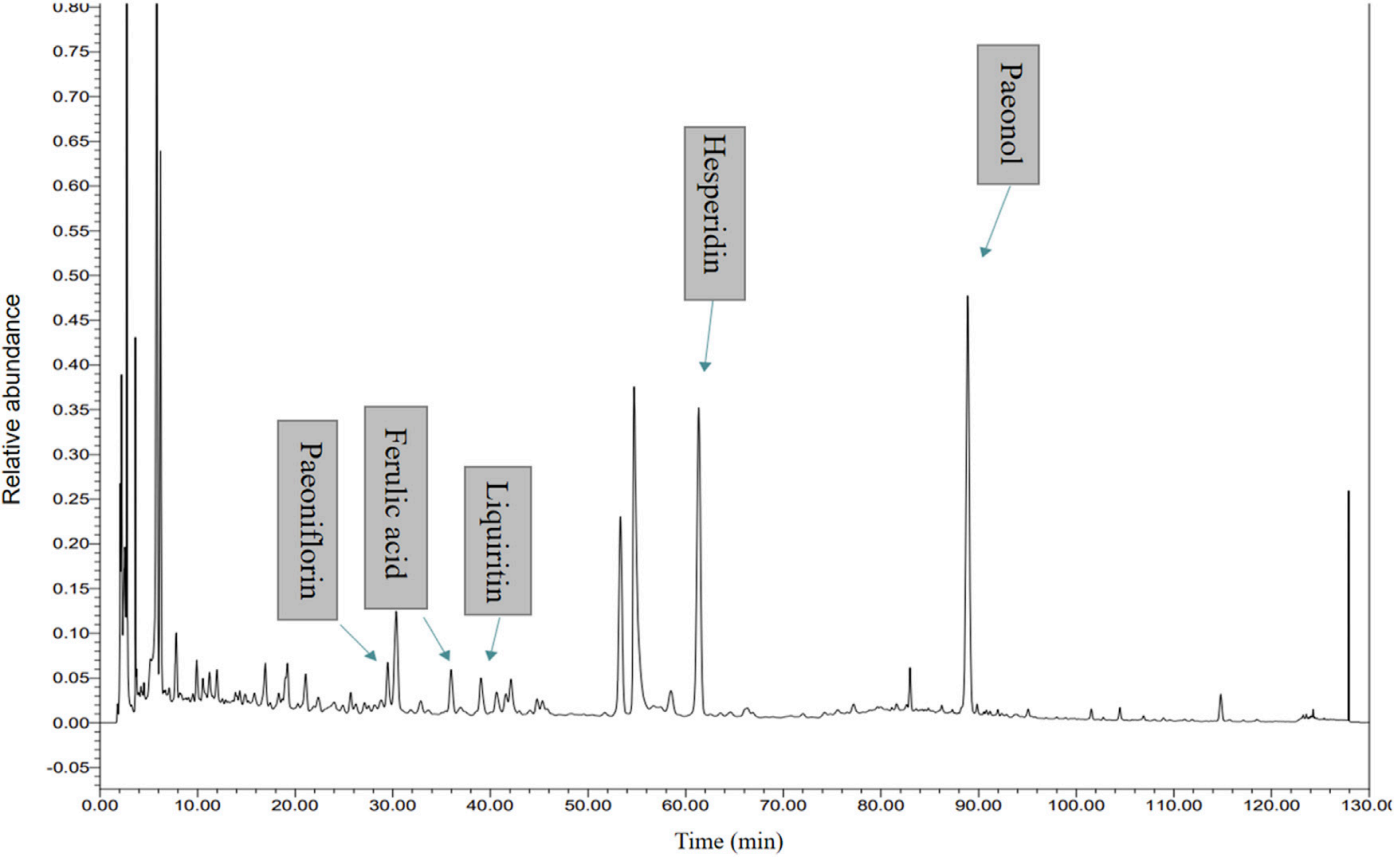
High Performance Liquid Chromatography (HPLC) analysis of ingredients from the Jingqianshu sample.

The quality control of the Jingqianshu granules was investigated using UHPLC-PDA. The UHPLC-PDA chromatogram of Jingqianshu is shown in Figure 3. Five components were identified: (1) paeoniflorin, (2) ferulic acid, (3) liquiritin, (4) hesperidin, and (5) paeonol.

### FST results after model building and screening

**FIGURE 4:**
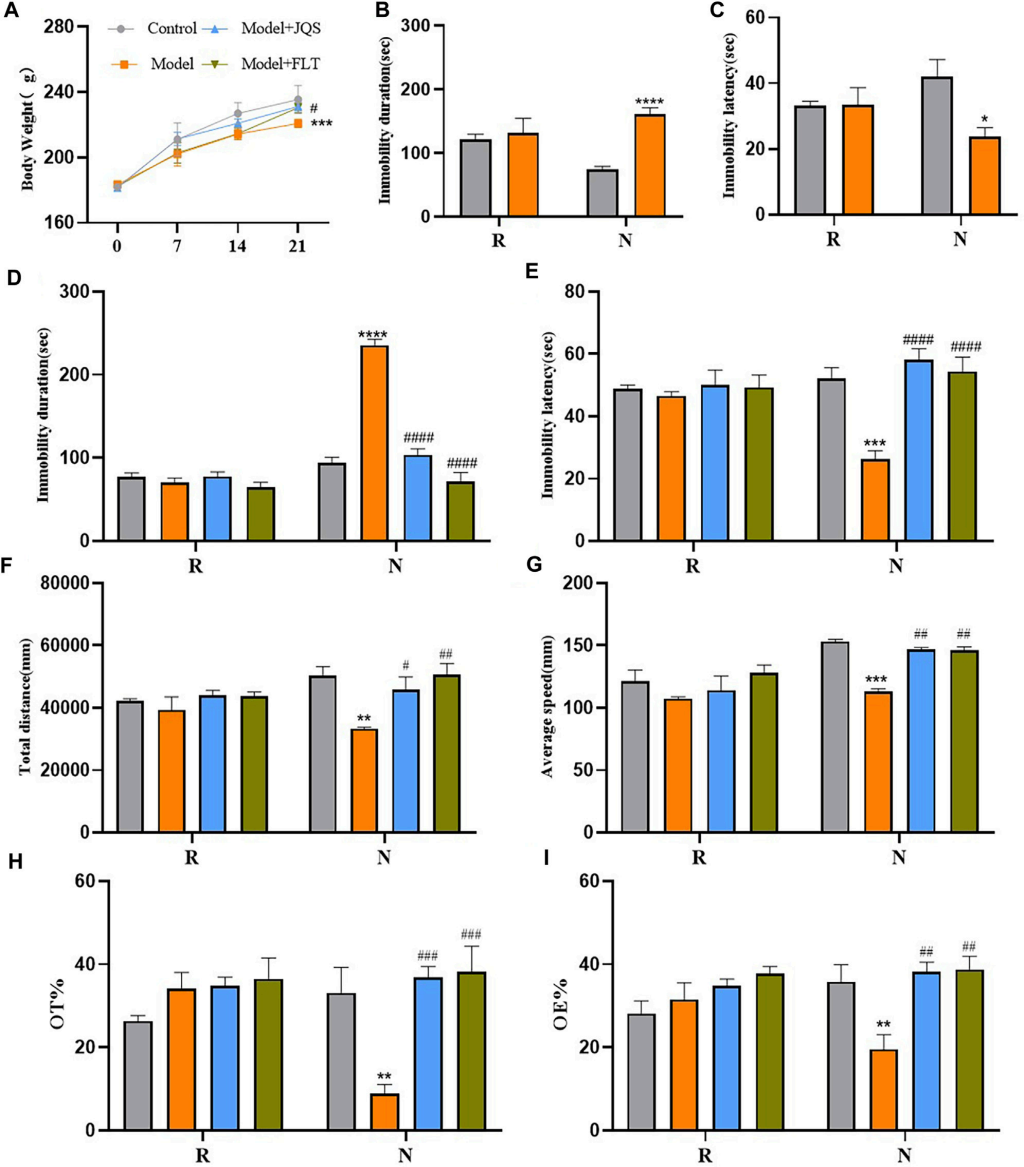
Weight and behavior tests. **(A)** Body Weight. **(B)** Immobility duration. **(C)** Immobility latency; **(D)** Immobility duration. **(E)** Immobility latency. **(F)** Total distance. **(G)** Average speed. **(H)** OT%. **(I)** OE%. N, the test in the non-receptive phase; R, the test in the receptive phase. *p < 0.05, **p < 0.01, ***p < 0.001, ****p < 0.0001 compared to the control group.^#^ *p* < 0.05, ^##^ *p* < 0.01, ^###^ *p* < 0.001, ^####^ *p* < 0.0001 compared to the model group; two-way ANOVA followed by post-hoc Sidak’s multiple comparisons test.

After dividing the experimental rats into control and model groups, the results of FST in the diestrus 1 (D1) phase (non-receiving phase [N]) and the proestrus/estrus (P/E) phase (receiving phase [R]) were analyzed. Analysis results showed that group factors had a significant effect on immobility duration (F_(1, 6)_ = 70.49, *p* = 0.0002) and immobility latency (F_(1, 6)_ = 6.330, *p* = 0.0455). During the N phase test, the rats in the model group were immobile for a longer duration (*p* < 0.0001) and had a shorter immobility latency (*p* < 0.05) compared with the control group (Figure 4B). However, there was no difference between the two groups in the R phase (Figure 4C).

### Jingqianshu granules improved premenstrual behavior in rats

Compared with the control group, the model group showed a significant decrease in body weight at day 21 (*p* = 0.0005), Jingqianshu granules (*p* = 0.0216) and fluoxetine (*p* = 0.038) reversed this phenomenon (Figure 4A). FST results are shown in Figures 4D, E, the group factor had an obvious effect on the immobility duration in the non-receptive phase (F_(3, 12)_ = 54.85, *p* < 0.0001), the model group had a longer immobility duration (*p* < 0.0001) compared to the control group, which was alleviated by Jingqianshu granules (*p* < 0.0001) and fluoxetine (*p* < 0.0001) interventions. Also, the same effect of group factor was observed for the immobility latency (F_(3, 12)_ = 14.70, *p* = 0.0003), with the model group showing a shorter latency (*p* < 0.0001) than the control group. Jingqianshu granules (*p* < 0.0001) and fluoxetine (*p* < 0.0001) reversed the shortening of immobility latency caused by FST. During the receptive phase, there was no difference between the four groups based on immobility duration and immobility latency.

The results of OFT are shown in Figures 4F, G, the group factor had a significant effect on total distance (F_(3, 12)_ = 7.393, *p* = 0.0046), and average speed (F_(3, 12)_ = 10.87, *p* = 0.0010). During the non-receiving phase, the model group showed a significantly shorter total distance (*p* = 0.0013) and decreased average speed (*p* = 0.0003) compared to the control group, and these changes returned to the control level after treatment with Jingqianshu granules (*p* = 0.0231, 0.0021, respectively) and fluoxetine (*p* = 0.0011, 0.0027, respectively). During the receptive phase, there were no differences in total distance and average speed among the four groups. In addition, the EPMT results are shown in Figures 4H, I. The group factor affected the OT% (F_(3, 12)_ = 7.003, *p* = 0.0056) and OE% (F_(3, 12)_ = 4.515, *p* = 0.0243) in the non-receptive phase, the OT% (*p* = 0.0019) and OE% (*p* = 0.0064) were lower in the model group than in the control group. Interestingly, both Jingqianshu granules and fluoxetine increased OT% (*p* = 0.0004, 0.0002, respectively) and OE% (*p* = 0.0016, 0.0012, respectively). There was no difference in OT% and OE% between the four groups during the receiving phase. The behavioral results suggest that a successful and reliable model of premenstrual depression was established using FST and that Jingqianshu granules have similar effects to fluoxetine in the treatment of premenstrual depression.

### Jingqianshu granules altered the expression of the orexin system

**FIGURE 5:**
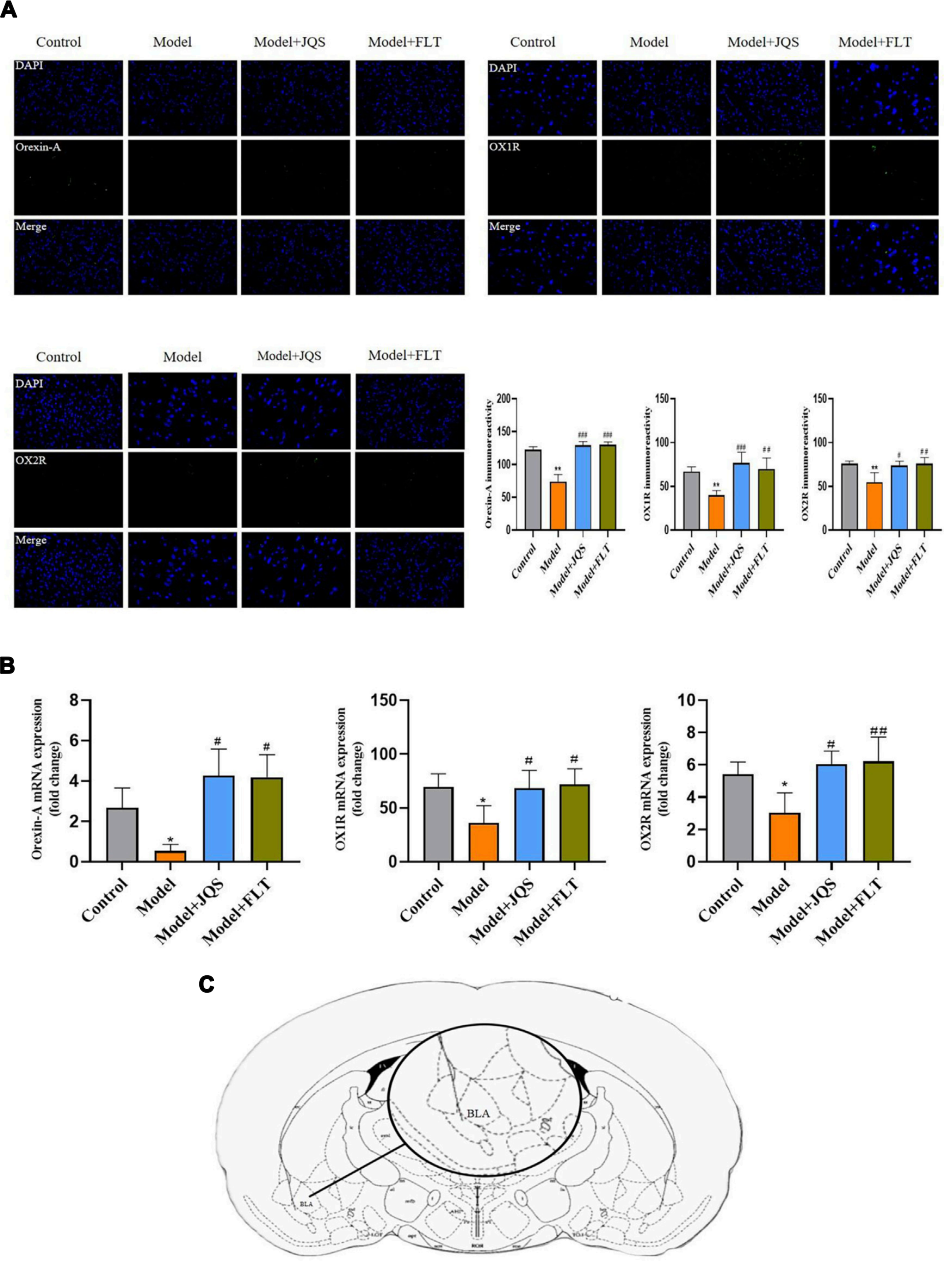
Immunofluorescence and RT-qPCR. **(A)** Measurement of immunoreactivity of orexin-A OX1R, and OX2R by immunofluorescence. **(B)** Detection of mRNA expression levels corresponding to orexin-A, OX1R, and OX2R proteins by RT-qPCR. **(C)** Brain regions examined. BLA, basolateral amygdala. *p < 0.05,**p < 0.01 compared to the control group. ^#^ *p* < 0.05, ^##^ *p* < 0.01, ^###^ *p* < 0.001 compared to the model group via one-way ANOVA.

Based on the immunofluorescence results (Figure 5A), the levels of orexin-A (F_(3, 12)_ = 15.76, *p* = 0.0002; control vs. model *p* = 0.0014), OX1R (F_(3,12)_ = 11.47, *p* = 0.0008; control vs. model *p* = 0.0085), and OX2R (F_(3,12)_ = 8.642, *p* = 0.0025; control vs. model *p* = 0.0054) were decreased in the model group compared with the control group, while the opposite was true for the rats treated with Jingqianshu granules (*p* = 0.0004, 0.0007, and 0.0105, respectively) and fluoxetine (*p* = 0.0004, 0.0039, and 0.0048, respectively). In addition, the mRNA levels of orexin-A (F_(3,12)_ = 12.92, *p* = 0.0120; control vs. model *p* = 0.033), OX1R (F_(3,12)_ = 5.188, *p* = 0.0158; control vs. model *p* = 0.0349), and OX2R (F_(3,12)_ = 6.848, *p* = 0.0061; control vs. model *p* = 0.0468) were decreased in the BLA brain region of the model rats (Figure 5C), compared with the control group, while the mRNA levels of the related proteins were increased after Jingqianshu granules (*p* = 0.0389, 0.0438, and 0.0122, respectively) and fluoxetine (*p* = 0.0127, 0.0235, and 0.0081, respectively) treatment (Figure 5B). These results indicate the protective effect exerted by Jingqianshu granules on orexin neurons, which may help to rescue premenstrually depressed rats.

### Jingqianshu granules regulate the expression of inflammatory factors

**FIGURE 6:**
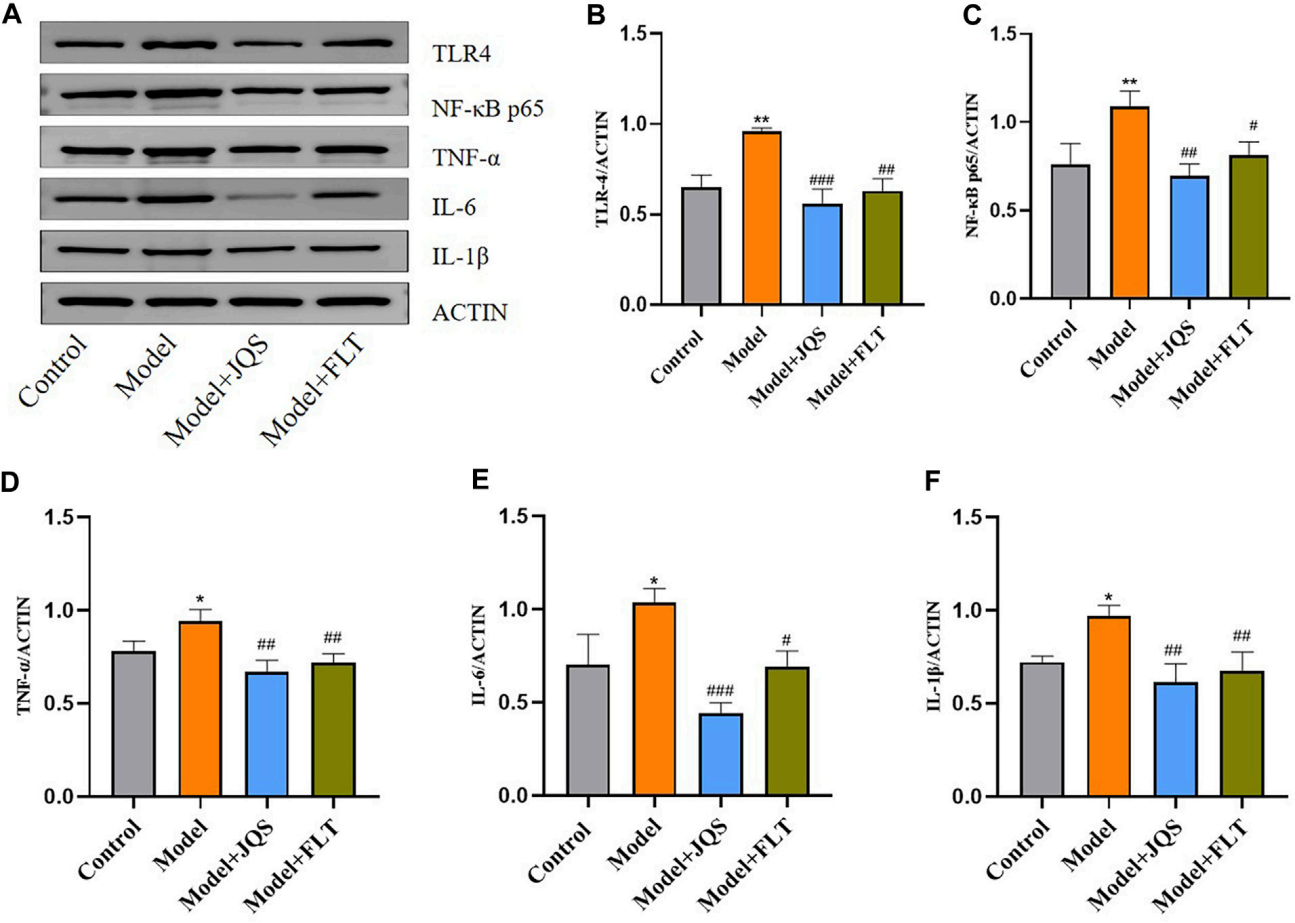
Protein expression of inflammatory factors after treatment with Jingqianshu granules. **(A)** Western blot for inflammatory factors. **(B)** TLR-4. **(C)** NF-κB p65. **(D)** TNF-α. **(E)** IL-6. **(F)** IL-1β. *p < 0.05,**p < 0.01 compared to the control group. ^#^ *p* < 0.05, ^##^ *p* < 0.01, ^###^ *p* < 0.001 compared to the model group via one-way ANOVA.

The immune response of Jingqianshu granules on brain regions of premenstrually depressed rats was examined by the levels of TLR4 (F_(3,8)_ = 23.24, *p* = 0.0003; control vs. model *p* = 0.0016), NF-κB (F_(3,8)_ = 11, *p* = 0.0033; control vs. model *p* = 0.0089), TNF-α (F_(3,8)_ = 12.96, *p* = 0.0019; control vs. model *p* = 0.0325), IL-6 (F_(3,8)_ = 16.75, *p* = 0.0008; control vs. model *p* = 0.0175), and IL-1β (F_(3,8)_ = 12.17, *p* = 0.0024; control vs. model *p* = 0.0187) (Figure 6). As shown, inflammatory factor protein levels were generally increased in the model rats compared to the control group, and Jingqianshu granules (*p* = 0.0003, 0.0032, 0.0017, 0.0005, 0.0023, respectively) and fluoxetine (*p* = 0.0011, 0.0239, 0.0059, 0.0149, 0.0068, respectively) restore these to control levels.

### Orexin-A improved premenstrual behavior in rats

**FIGURE 7:**
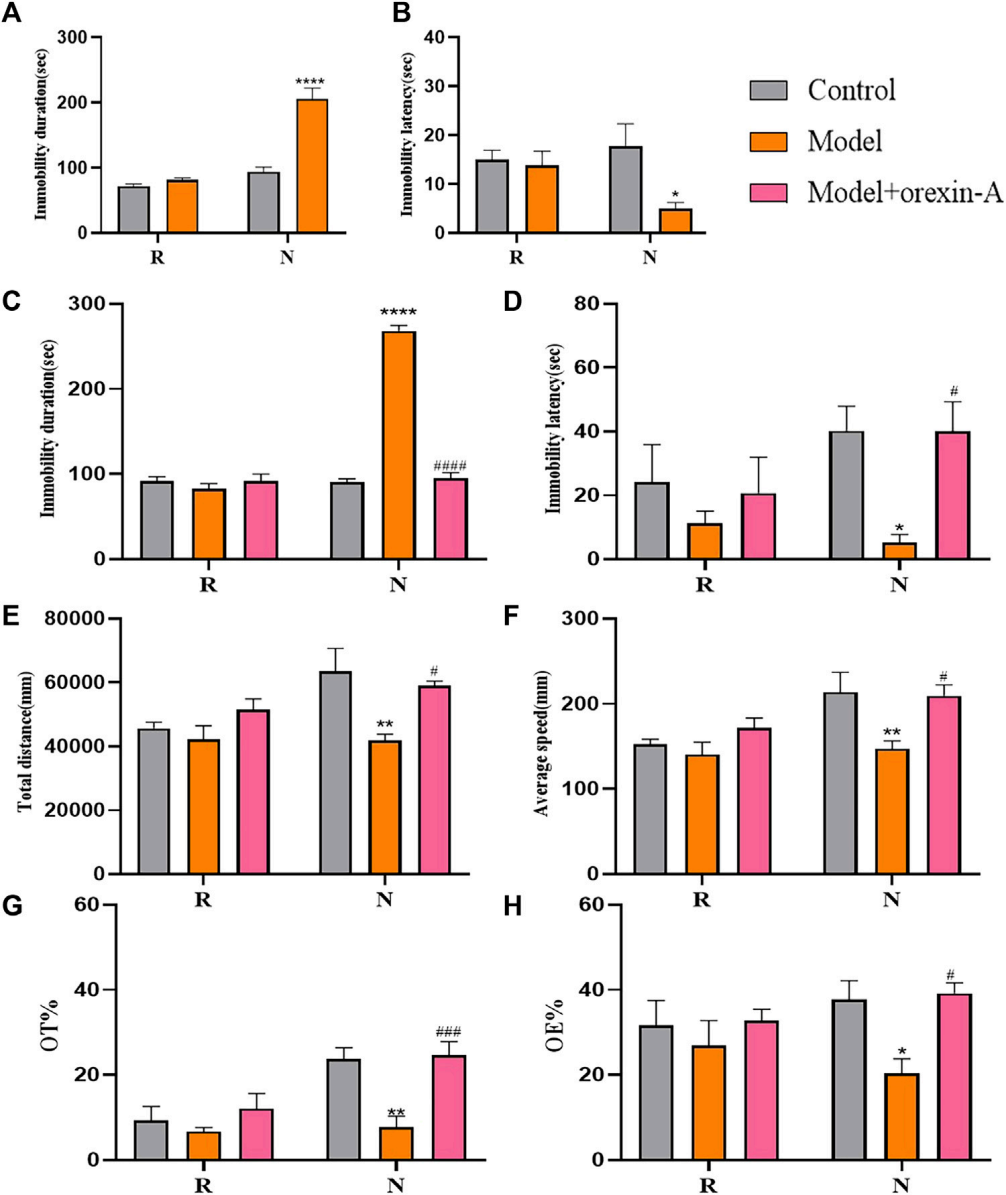
Behavior tests. **(A)** Immobility duration. **(B)** Immobility latency. **(C)** Immobility duration. **(D)** Immobility duration. **(E)** Total distance. **(F)**Average speed. **(G)** OT%. **(H)** OE%. N, the test in the non-receptive phase; R, the test in the receptive phase. *p < 0.05, **p < 0.01, ***p < 0.001, ****p < 0.0001 compared to the control group. ^#^ *p* < 0.05, ^##^ *p* < 0.01, ^###^ *p* < 0.001, ^####^ *p* < 0.0001 compared to the model group, two-way ANOVA followed by post-hoc Sidak’s multiple comparisons test.

The effect of orexin-A injection in the BLA brain region on rat behavior is shown in Figure 7. The group factor had an obvious effect on the immobility duration in the non-receptive phase (F_(2, 15)_ = 81.98, *p* < 0.0001), which was longer in the model group than in the control group (*p* < 0.0001), which was alleviated by orexin-A (*p* < 0.0001) intervention. Meanwhile, the same effect of group factor was observed for the immobility latency (F_(2, 9)_ = 5.457, *p* = 0.0280) with a lower value in the model group (*p* = 0.0269) compared to that in the control group, treatment with orexin-A (*p* = 0.0275) reversed this effect. During the receptive phase, there was no difference between the three groups according to the duration of immobility and the latency of immobility. Similarly, the group factor had a significant effect on total distance (F_(2, 15)_ = 6.692, *p* = 0.0084) and average speed (F_(2, 15)_ = 5.537, *p* = 0.0158). During the non-receptive phase, the total distance was significantly shorter (*p* = 0.0013) and the average speed decreased (*p* = 0.0061) in the model group compared to the control group, and these changes returned to control levels after orexin-A treatment (*p* = 0.0121, 0.0108, respectively). There was no difference in total distance or average speed between the three groups during the receptive phase. In addition, the group factor affected the OT% (F_(2, 15)_ = 9.974, *p* = 0.0018) and OE% (F_(2, 9)_ = 4.961, *p* = 0.0353) in the non-receptive phase, EPM results showed that OT% (*p* = 0.0012) and OE% (*p* = 0.0346) were lower in the model group than in the control group. Interestingly, orexin-A increased OT% (*p* = 0.0006) and OE% (*p* = 0.0201). There was no difference in OT% and OE% between the three groups during the receptive phase. The above behavioral evidence suggests a positive role of orexin-A in the treatment of premenstrual depressive disorders.

### Orexin-A treatment attenuates microglia activation

**FIGURE 8:**
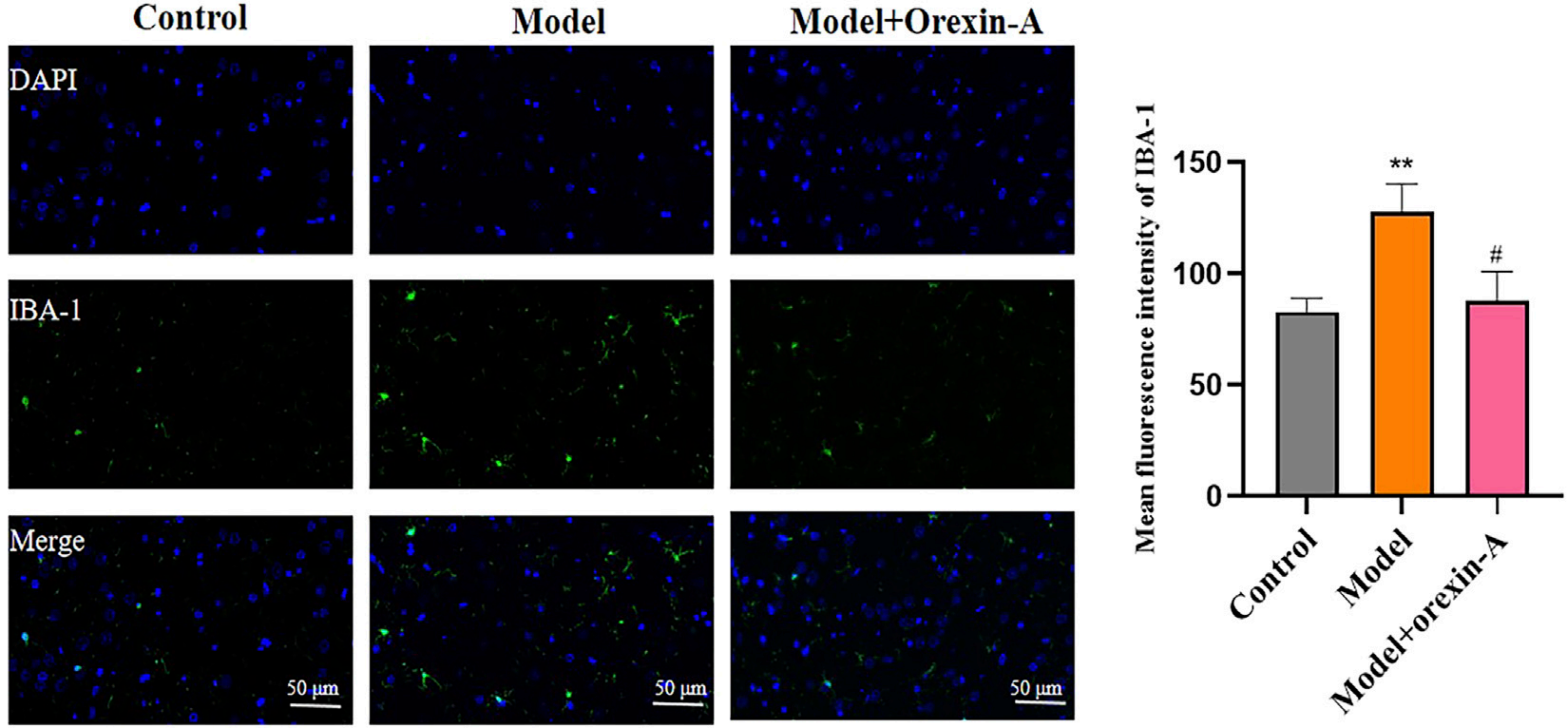
Measurement of immunoreactivity of IBA-1, orexin-A inhibits microglia cell activation.

We used IBA-1 markers in immunofluorescence to assess the microglia activation status in BLA brain regions of rats, the results are shown in Figure 8. Compared with the control group, the model group of rats showed increased IBA-1 immunoreactivity (F_(2,6)_ = 14.29, *p* = 0.0052; control vs. model *p* = 0.0067). And orexin-A (*p* = 0.0115) treatment reduced the area of IBA-1staining, thus reducing microglia activation.

### Orexin-A reverses the increase in TLR-4 expression and activation of NF-κB signaling in BLA brain regions

**FIGURE 9:**
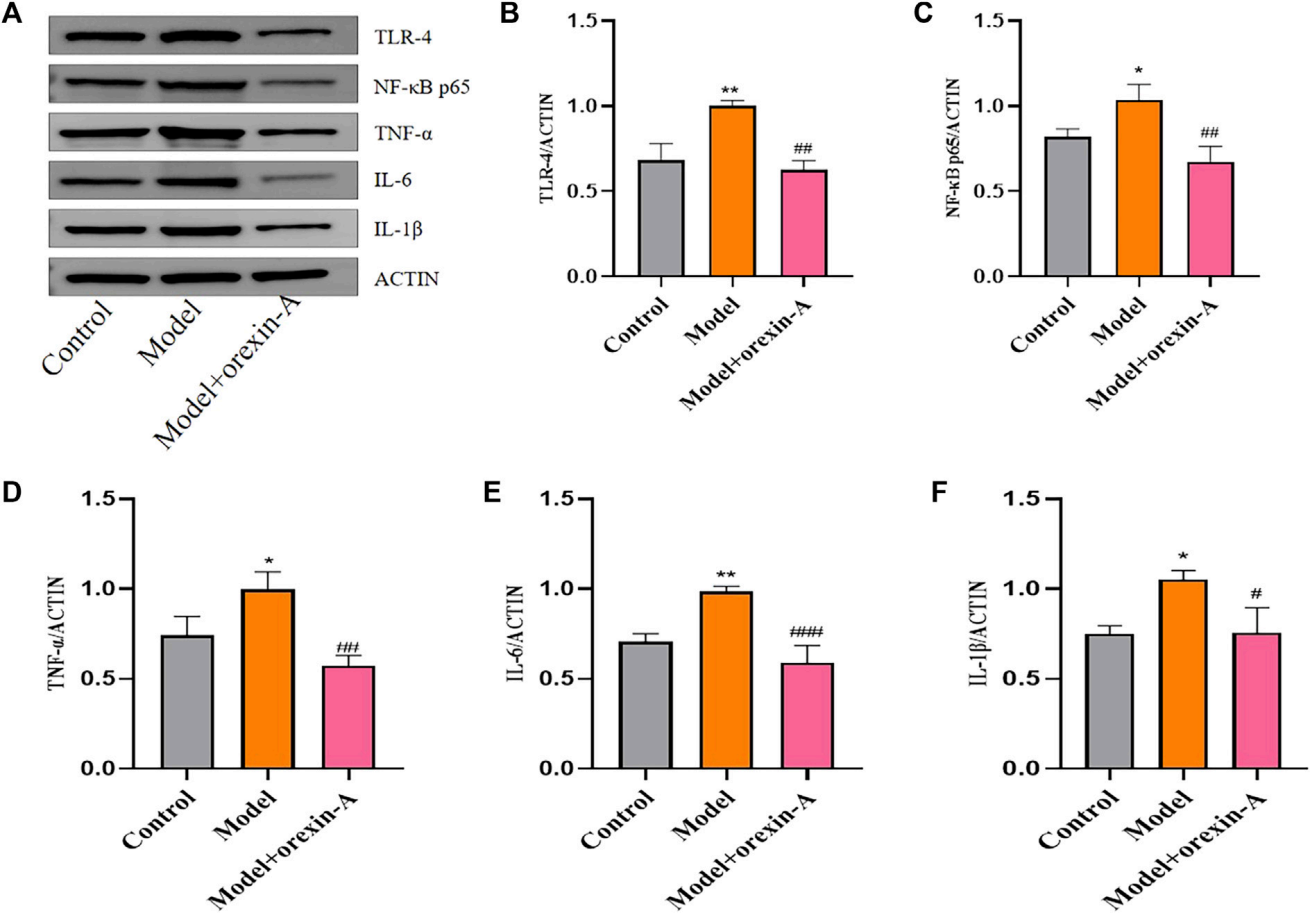
Protein expression of inflammatory factors after treatment with orexin-A. **(A)** Western blot for inflammatory factors. **(B)** TLR-4. **(C)** NF-κB p65. **(D)** TNF-α. **(E)** IL-6. **(F)** IL-1β. *p < 0.05,**p < 0.01 compared to the control group. ^#^ *p* < 0.05, ^##^ *p* < 0.01, ^###^ *p* < 0.001 compared to the model group via one-way ANOVA.

Toll-like receptors have a regulatory role in the type of acquired immune response. Activation of most TLRs induces anti-microbial defense systems that produce IL-1β, IL-6, and TNF pro-inflammatory cytokines (Squillace and Salvemini, 2022). NF-κB is a common regulator of cytokine production and can be activated by TLR signaling and other pro-inflammatory stimuli. Therefore, we investigated the expression of TLR4 and NF-κB as well as downstream signaling. Analysis of western blot results showed that TLR4 (F_(2,6)_ = 27.16, *p* = 0.0010; control vs. model *p* = 0.0028), NF-κB (F_(2,6)_ = 15.76, *p* = 0.0041; control vs. model *p* = 0.0355), TNF-α (F_(2,6)_ = 17.50, *p* = 0.0031; control vs. model *p* = 0.0282), IL-6 (F_(2,6)_ = 30.68, *p* = 0.0007; control vs. model *p* = 0.0041), and IL-1β (F_(2,6)_ = 11.30, *p* = 0.0092; control vs. model *p* = 0.0141) protein levels were increased in model rats compared with controls, and orexin-A (*p* = 0.0012, 0.0034, 0.0026, 0.0007, 0.0154, respectively) intervention reduced the expression levels of these factors (Figure 9).

## Discussion

In this study, an FST-based model of premenstrual depression was developed in which model rats exhibited a range of depressive-like behaviors. During the non-receptive phase, with prolonged immobility and shortened immobility latency in the FST, similarly shorter total distance and lower average speed in the OFT, and reduced time and number of entries into the open arm, which disappeared during the receptive phase. In Part 1 of the experiment, behavioral tests and the detection of orexin system and inflammatory factors found that Jingqianshu granules reversed behavioral symptoms and changes in the levels of orexin and inflammatory signals TLR4, NF-κB, TNF-α, IL-6, and IL-1β. In Part 2 of the experiment, through behavioral testing and detection of microglia and inflammatory factor levels, orexin-A was found to inhibit microglia activation and reverse changes in inflammatory factor levels such as TLR4, NF-κB, TNF-α, IL-6, and IL-1β.

Orexin was first identified as a neuropeptide in the hypothalamus, which is associated with feeding behavior (Tsujino and Sakurai, 2009) and regulation of sleep arousal (Sakurai, 2007) and acts through the hypothalamus projections into multiple brain regions. The BLA receives direct fiber projections from the lateral hypothalamic region and releases orexin, which regulates the activity of excitatory, inhibitory neuronal loops in the BLA by activating presynaptic OX1R (Tsunematsu and Yamanaka, 2012). It is significant that during the experiment we found the expression of orexin and its receptor in the rat BLA brain region. A recent systematic review of studies conducted over the past 15 years indicated that orexin was associated with depressive behavior, and scientists have found that orexin levels varied along with depression-like behavior (Khairuddin et al., 2020). In addition to depression, orexin also has been associated with several other disorders that coexist with depression, including narcolepsy, addiction, and appetite disorders. Dysregulation of the orexin system is an important mechanism involved in the onset of depressive symptoms. Depressed patients with concomitant suicidal tendencies had lower serum orexin-A, compared with normal subjects (Brundin et al., 2007). It was found that serum orexin-B levels were lower in depressed patients than in normal controls and that the orexin-B levels and HAMD scores were significantly negatively correlated. Similarly, orexin-B levels were significantly higher at the end of 4 and 8 weeks of treatment, with statistically significant differences (Salomon et al., 2003). The relationship between neurotransmission of the orexin system and depression has also been confirmed in a rat model of depression. The abnormal changes of orexin-A/OX1R in the lateral hypothalamus of chronic stress-induced depressed rats were found to be closely associated with the pathogenesis of depression with somatic symptoms (Hou et al., 2020). Based on the above reports and the similarity between the microscopic mechanisms of PMDD and MDD, we hypothesized that the pathogenesis of PMDD is related to a malfunction of the orexin system. The experimental results demonstrated reduced levels of orexin, OX1R, and OX2R genes and proteins of rats in premenstrual depression model, which were restored to the control levels after drug intervention. Thus, orexin may be one of the targets for premenstrual depression treatment.

It was found that mood-related behaviors may also depend on the stage of the estrous cycle, and differences in the amygdala may be one of the reasons for this (Blume et al., 2017). During the menstrual cycle, amygdala responses correlate with progesterone levels. And, amygdala reactivity in PMDD patients may be more sensitive than subjective scores. This parallels the increased reactivity of the amygdala to emotional stimuli (Gingnell et al., 2014). The BLA is a core brain region that integrates sensory information from cortical and subcortical afferents into glutamatergic projection neurons (PNs) and transmits them to downstream brain structures responsible for the expression of emotions, such as depressive behavior (Hakamata et al., 2022). Therefore, we investigated the expression of orexin in the BLA brain region of premenstrually depressed rats. The results reveal that the pathomechanism of PMDD is related to the dysfunction of BLA.

Interestingly, we found that inflammatory factor expression was elevated in premenstrually depressed rats and decreased after treatment. Abnormalities in inflammatory cytokines have been reported to be associated with the pathogenesis of PMDD, and the interaction of orexin with inflammatory factors has been demonstrated. In an investigation of the relationship between changes in physical and psychological symptoms and inflammatory markers in normal-weight and overweight women during the menstrual cycle, Puder et al. found that changes in TNF-α and hs-CRP serum concentrations were associated with physical and psychological symptoms of the menstrual cycle (Puder et al., 2006). In a study of the relationship between inflammatory markers and the severity of menstrual symptoms and PMS in young women, mean levels of IL-4, IL-10, IL-12, and IFN-g were found to be significantly higher in women meeting PMS criteria than in healthy women (Bertone-Johnson et al., 2014). In a review of the epidemiology and treatment of PMDD, Hantsoo et al. indicated that inflammatory factors may play a role in the pathology of PMDD and that a study conducted in women with premenstrual symptoms showed an increase in pro-inflammatory markers compared to controls (Hantsoo and Epperson, 2015). There appears to be sufficient preliminary evidence for a link between neuroinflammation and the etiology of PMDD (Bannister, 2019; Gold et al., 2016). Modi et al. using intranasal Orexin in a rat model of cardiac arrest (CA) found that CA increased pro-inflammatory markers in all brain regions and that ORXA treatment significantly improved CA-induced neuroinflammatory markers in the hypothalamus, increased the production of orexin receptors (ORX1R and ORX2R), had anti-inflammatory effects and accelerated cortical EEG and behavioral recovery (Modi et al., 2017). In his experiments, Xu et al. found that orexin reduced the inflammatory response in cerebral ischemia/reperfusion injury by inhibiting the levels of IL-1β, TNF-α, and IL-6 inflammatory factors (Xu et al., 2021). We wondered whether Jingqianshu granules might regulate the coordinated action of orexin with inflammatory factors to treat premenstrual depression. To confirm this conjecture, we used orexin injections in the brain and found that orexin-A alleviated depressive-like behavior and inhibited microglia activation and TLR production, thereby inhibiting the activation of the NF-κB signaling pathway and reducing the release of inflammatory factors.

However, our study has certain limitations. Firstly, Of the five components identified in the Jingqianshu granules, paeoniflorin is involved in upregulating monoaminergic neurotransmitter levels, suppressing hypothalamic-pituitary-adrenal axis hyperfunction, and having broad immune and anti-inflammatory effects (Zhang and Wei, 2020); ferulic acid has anti-inflammatory and antioxidant effects (Zduńska et al., 2018); liquiriti affects neuro-endocrine-immune network regulation (Lan et al., 2020); hesperidin is involved in hippocampal neurotrophic factor regulation and inhibition of inflammatory response (Xie et al., 2020); paeonol can inhibit the inflammatory response and is involved in neurotransmitter regulation (Tao et al., 2016). All five chemical components have been shown in separate studies to be effective in alleviating depressive symptoms. However, little is known about the effects of the orexin system. Jingqianshu granules can effectively modulate the orexin system to alleviate premenstrual depression, which may be related to the synergistic effects of their complex drug components. The specific mechanism of chemical components and their derivatives involved in orexin regulation remains to be investigated. Secondly, Fluoxetine is used as a first-line drug for PMDD treatment, and Jingqianshu granules show similar effects to it. However, due to the limited experimental sample size and short treatment time, there was no observed change of adverse side effects after fluoxetine was given to rats. Therefore, long-term therapeutic studies are necessary. Once again, it is difficult to fully reproduce the symptoms of PMDD patients in an animal model. We followed previous studies to establish an FST-based model of premenstrual depression under the condition of ensuring a normal estrous cycle in rats. However, due to the complex pathogenesis of PMDD and possible differences in pathology, physiology, and drug resistance between animal models and patients, more in-depth exploration is needed.

In conclusion, our findings suggest that orexin system dysfunction may be a potential pathological mechanism leading to depression-related symptoms and that Jingqianshu granules may achieve the treatment of premenstrual depression by modulating orexin-A-mediated inflammatory factors, providing new insights for further research into the pathogenesis of PMDD. Of course, the present study is preliminary, and the pathogenesis of PMDD is complex. So more in-depth exploration is needed, such as the evaluation of the downstream molecular signaling pathways of orexin or the metabolic mechanism of orexin in the brain. We will explore this further in the future.

## Data Availability

The original contributions presented in the study are included in the article/Supplementary Material, further inquiries can be directed to the corresponding authors.
