# Supplementary material for: Jingqianshu granules mitigates premenstrual depression by regulating orexin signaling
Source: Front Pharmacol. 2024 Jun 14;15:1294122. doi: 10.3389/fphar.2024.1294122 (PMC11211579; doi:10.3389/fphar.2024.1294122)
Supplement: Supplementary file 1 [file Table1.DOCX]

**Supplemental Table 1.** PCR primers used in this study

| Gene | Forword (5’→3’) | Reverse (5’→3’) |
| --- | --- | --- |
| Orexin-A | TCCTGCCGTCTCTACGAACTGT | TGGTTACCGTTGGCCTGAA |
| OX1R | AACAGTGCGGCCAACCCTAT | ACCGGCTCTGCAAGGACAAG |
| OX2R | TCGAAACAGCATCGTTGTCATCT | CCATGTACGTCACCAGAAAGAAGC |
| GAPDH | CTGGAGAAACCTGCCAAGTATG | GGTGGAAGAATGGGAGTTGCT |
